# Supplementary material for: Anhydrous interfacial polymerization of sub-1 Å sieving polyamide membrane
Source: Nat Commun. 2023 Nov 22;14:7624. doi: 10.1038/s41467-023-43291-2 (PMC10665378; doi:10.1038/s41467-023-43291-2)
Supplement: Supplementary file 1 — Supplementary Information [file 41467_2023_43291_MOESM1_ESM.pdf]

**Supplementary Information**

# Anhydrous interfacial polymerization of sub-1 Å sieving Polyamide membrane

Guangjin Zhao [1], Haiqi Gao[2], Zhou Qu[1], Hongwei Fan[1*], and Hong Meng[2*]

[1] College of Chemical Engineering, Beijing University of Chemical Technology, Beijing, 100029, PR China. Email: fanhongwei@mail.buct.edu.cn

[2] State Key Laboratory of Chemistry and Utilization of Carbon Based Energy Resources, College of Chemistry, Xinjiang University, Urumqi, 830046, PR China. Email: menghong@xju.edu.cn

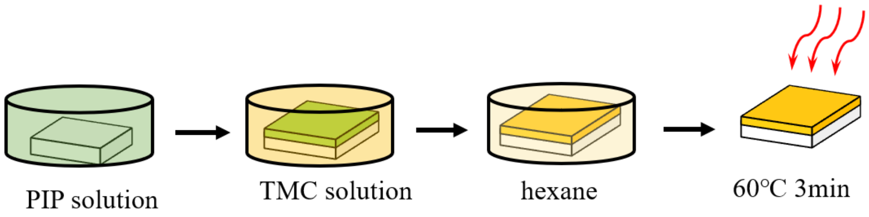


**Supplementary Figure 1.** A schematic illustration showing the preparation process of CIP-PA membrane.

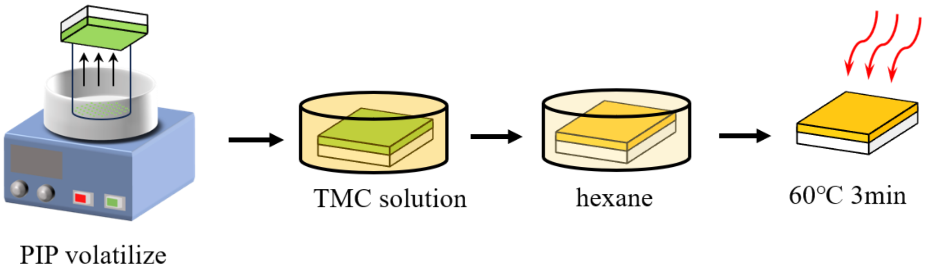


**Supplementary Figure 2.** A schematic illustration showing the preparation process of AIP-PA membrane.

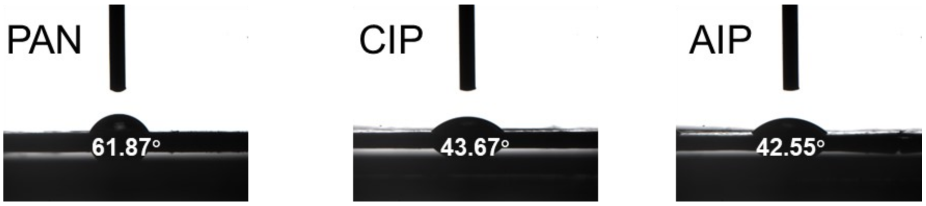


**Supplementary Figure 3.** Water contact angle of PAN substrate and PA membranes.

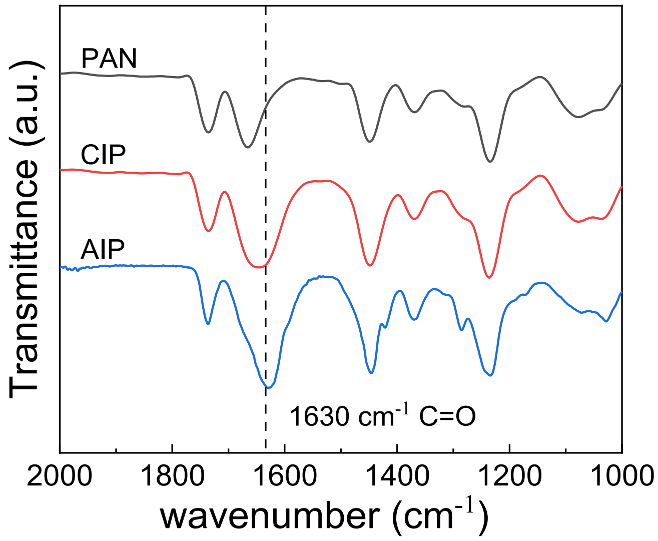


**Supplementary Figure 4.** ATR-FTIR survey of PAN substrate and PA membranes.

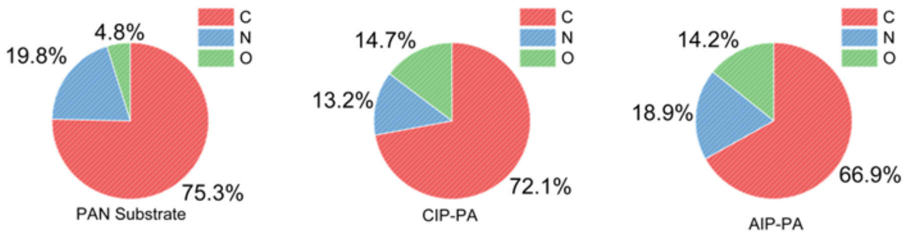


**Supplementary Figure 5.** The elements contents of PAN substrate and PA membranes.

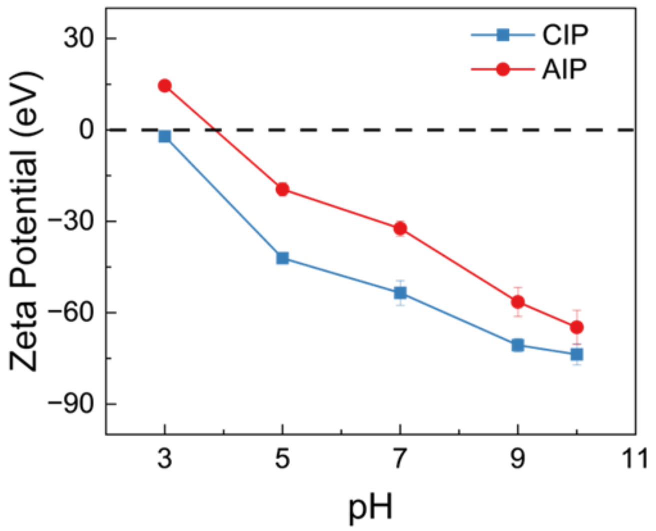


**Supplementary Figure 6.** Zeta potential of PA membranes.

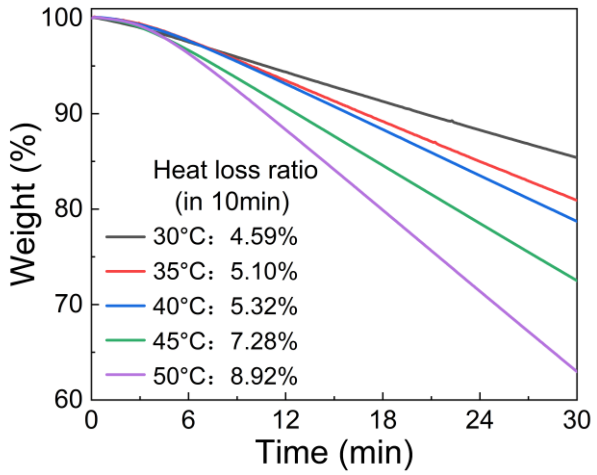


**Supplementary Figure 7.** Mass volatilization curves of PIP at different temperatures.

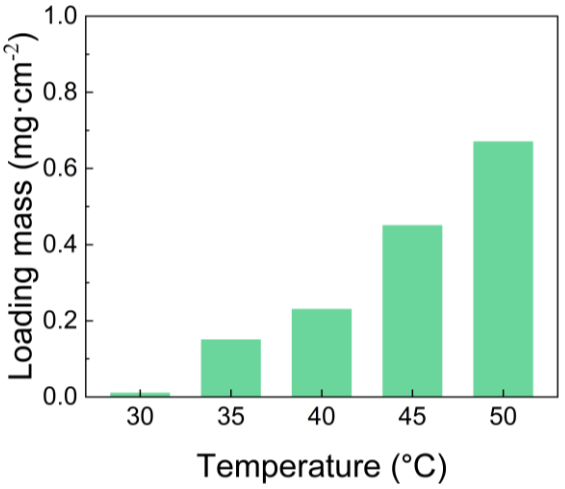


**Supplementary Figure 8.** PIP loading mass adsorbed on the substrate surface at different temperatures.

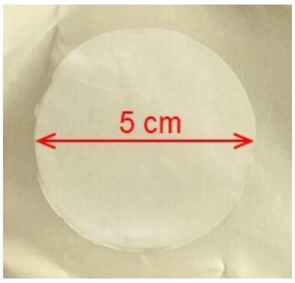


**Supplementary Figure 9.** Uniformity of PIP molecules on the surface of the tin.

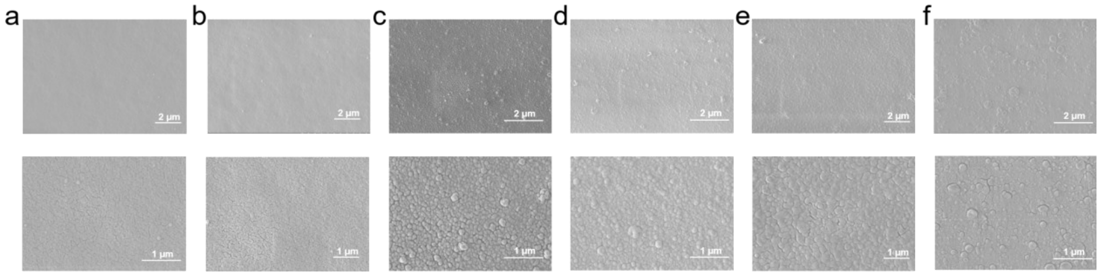


**Supplementary Figure 10.** SEM image of AIP membranes. (a)PAN substrate, (b) AIP-PA@30, (c) AIP-PA@35, (d) AIP-PA@40, (e) AIP-PA@45, (f) AIP-PA@50.

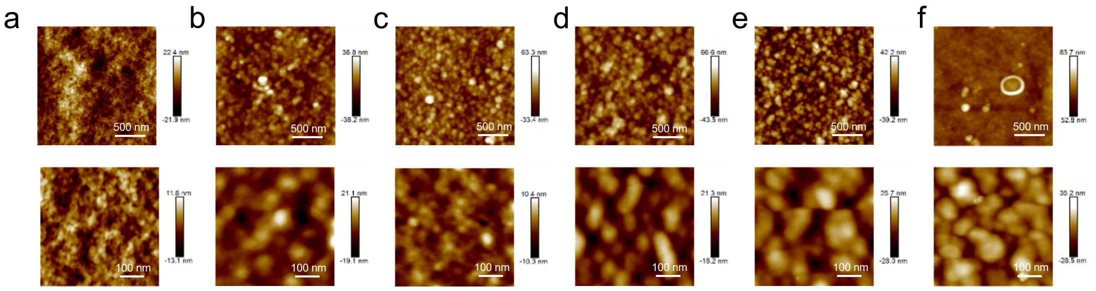


**Supplementary Figure 11.** 2D AFM image of AIP membranes. (a)PAN substrate, (b) AIP-PA@30, (c) AIP-PA@35, (d) AIP-PA@40, (e) AIP-PA@45, (f) AIP-PA@50.

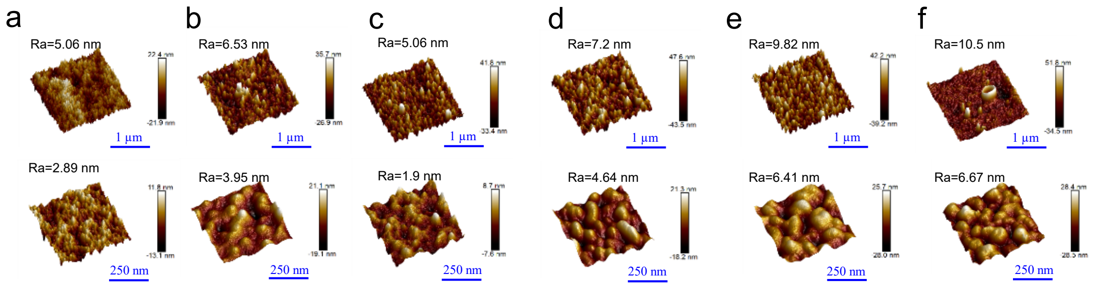


**Supplementary Figure 12.** 3D AFM image of AIP membranes. (a)PAN substrate, (b) AIP-PA@30, (c) AIP-PA@35, (d) AIP-PA@40, (e) AIP-PA@45, (f) AIP-PA@50.

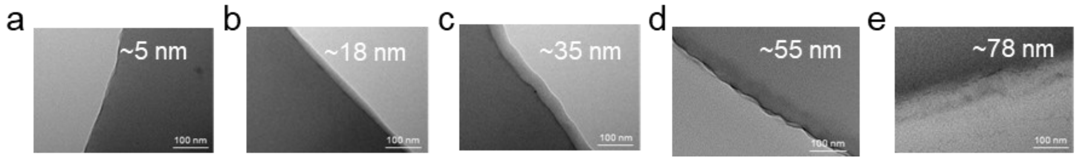


**Supplementary Figure 13.** TEM image of AIP-PA membranes. (a) AIP-PA@30, (b) AIP-PA@35, (c) AIP-PA@40, (d) AIP-PA@45, (e) AIP-PA@50.

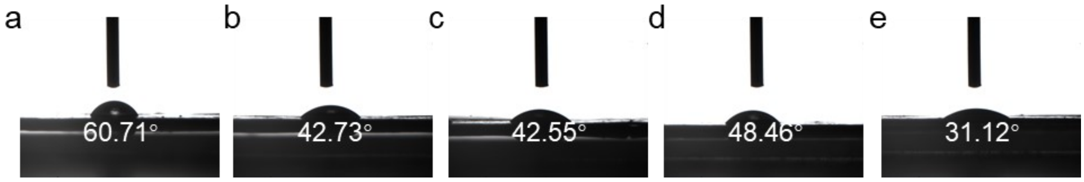


**Supplementary Figure 14.** Water contact angle of AIP-PA membranes at different temperature. (a) AIP-PA@30, (b) AIP-PA@35, (c) AIP-PA@40, (d) AIP-PA@45, (e) AIP-PA@50.

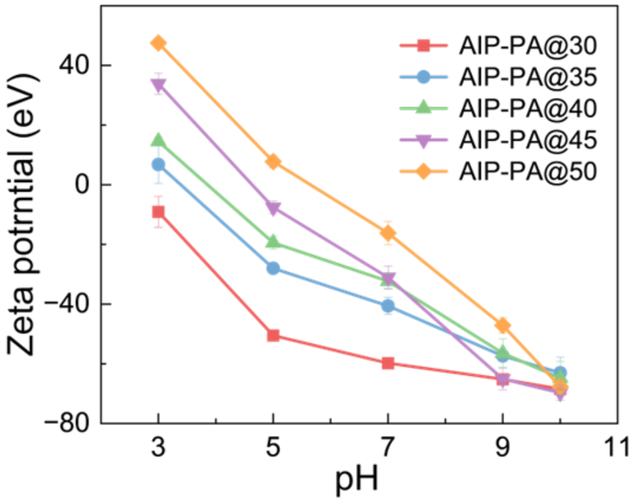


**Supplementary Figure 15.** Zeta Potential of PA membranes fabricated via AIP-PA at different temperature.

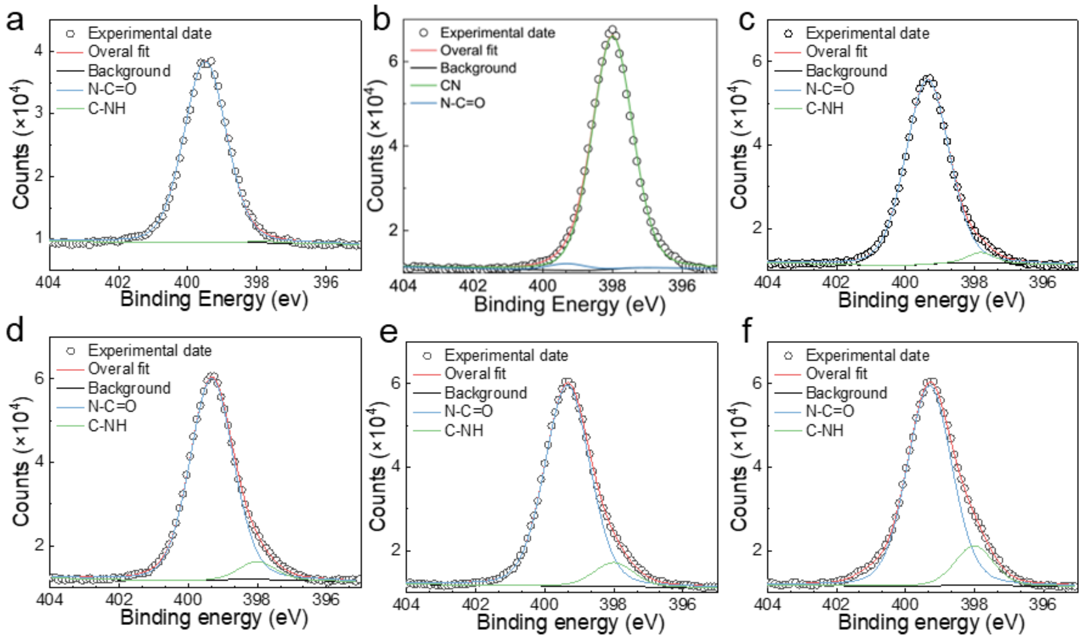


**Supplementary Figure 16.** High-resolution XPS spectra of N (1*s*) of AIP-PA and CIP-PA membranes. (a) CIP-PA, (b) AIP-PA@30, (c) AIP-PA@35, (d) AIP-PA@40, (e) AIP-PA@45, (f) AIP-PA@50.

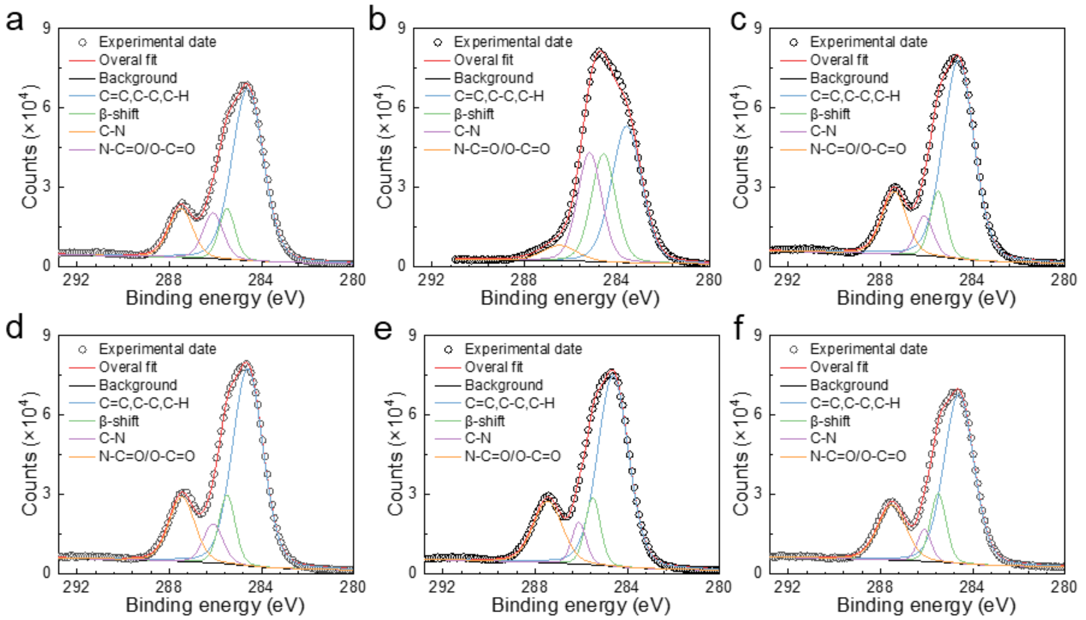


**Supplementary Figure 17.** High-resolution XPS spectra of C (1*s*) of AIP-PA and CIP-PA membranes. (a) CIP-PA, (b) AIP-PA@30, (c) AIP-PA@35, (d) AIP-PA@40, (e) AIP-PA@45, (f) AIP-PA@50.

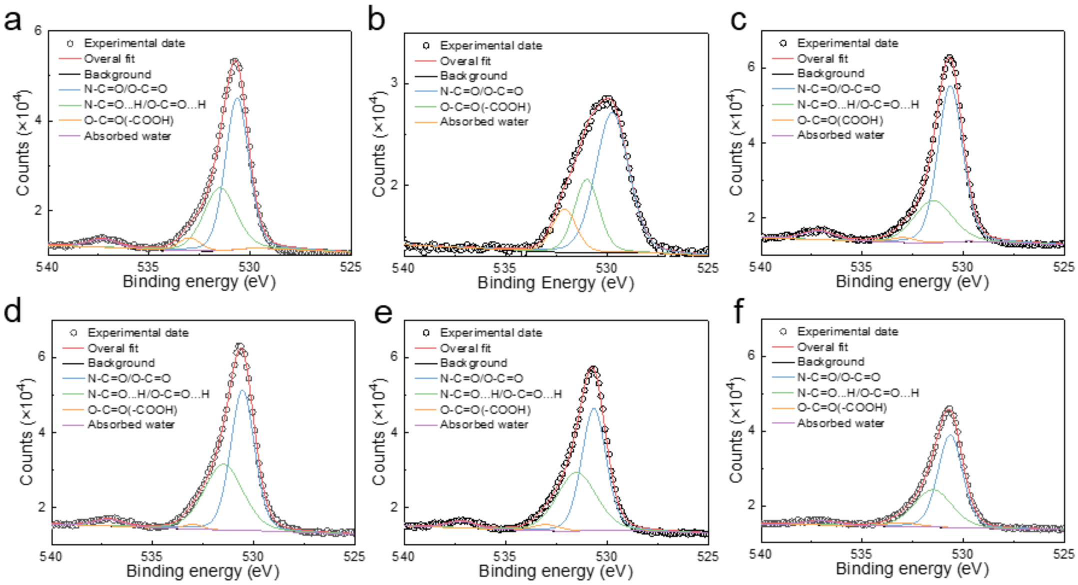


**Supplementary Figure 18.** High-resolution XPS spectra of O (1*s*) of AIP-PA and CIP-PA membranes. (a) CIP-PA, (b) AIP-PA@30, (c) AIP-PA@35, (d) AIP-PA@40, (e) AIP-PA@45, (f) AIP-PA@50.

The information of chemical bonding was obtained by deconvolution of C (1*s*), N (1*s*) and O (1*s*) high resolution XPS spectra as shown in Supplementary Fig. 16-18 and Supplementary Table 1-4. The C (1*s*) high resolution spectra for all membranes show three peaks. However, the surface chemistry of the AIP-PA@30 membrane is different from other membranes due to the presence of defects on the surface, which leads to the detection of the chemical bonding of the PAN substrate. For example, the appearance of C≡N bonds at 286.5 eV, the C-N bonds at 285.2ev and the C-H bonds at 283.6 eV. For other defect-free PA membranes, the main peak at 284.6 eV is attributed to carbon atoms without adjacent electron-withdrawing atoms (C in C-C or C-H bonds); A β-shift (ΔE≈+0.7 eV) was attributed to the C1s photoelectron from the aromatic carbon of the TMC, which are connected either to the amide (C-C(-N)=O) or carboxylic acid group (C-C(=O)OH). The middle peak at 285.7 eV is related to the weakly electron-withdrawing carbon atom (C in the C-N bond); The small peak at 287.45 eV can be attributed to the carbon attached to the strong electron-withdrawing atom (O=C-O in the carboxyl group and O=C-N in the amide). The O (1*s*) high resolution spectra for all membranes also show three peaks, indicating three types of oxygen are exited in the PA layer: The O=C-N bond at 530.65 eV, N-C=O…H/O-C=O…H bond at 531.5 eV, O-C=O bond at 533 eV and the oxygen in the adsorbed water at 537.2 eV. The N (1*s*) high resolution spectra for all membranes show two peaks. However, the surface chemistry of the AIP-

PA@30 membrane is different from other membranes due to the presence of defects on the surface, which leads to the detection of -CN bonds at 398 eV of the PAN substrate. For other defect-free PA membranes, there are two types of oxygen are exited in the PA layer: the N-C=O bond at 399.4 eV and the C-NH bond at 398 eV.

In addition, XPS was also used to examine the surface element changes of the prepared PA membranes with varying amounts of volatilization. The C-N content grew first and then reduced in the deconvolution of C1*s*, while the -COOH content decreased first and then increased in the deconvolution of O1*s*, with the extreme points all at 40 °C, showing that the synthesized PA membrane had the best cross-linking characteristics at this moment. The deconvolution of N1*s* shows that the PA membrane's surface accumulates more C-NH as the more the amine monomer is volatilized.

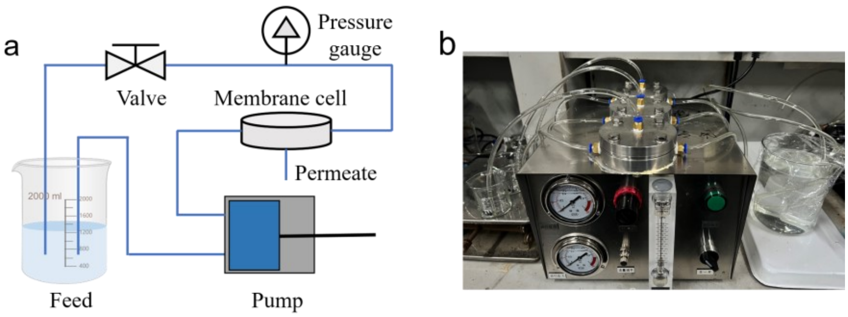


**Supplementary Figure 19.** (a) Cross-flow nanofiltration process and (b) corresponding digital photograph of the device.

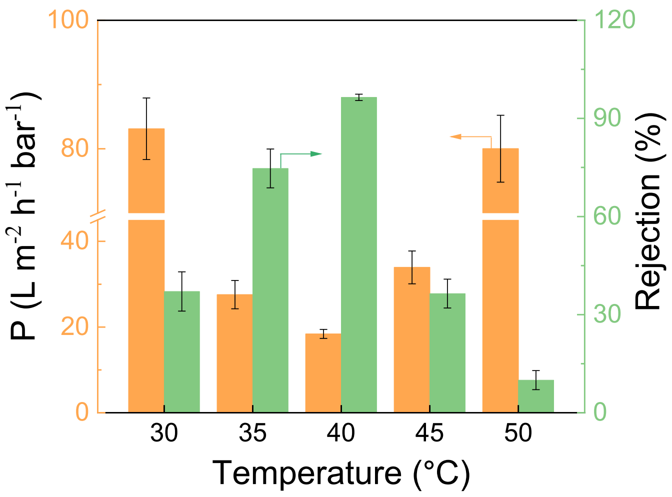


**Supplementary Figure 20.** The effect of volatilization temperature on NF performance. The error bars represent the standard deviation of data from three replicate measurements.

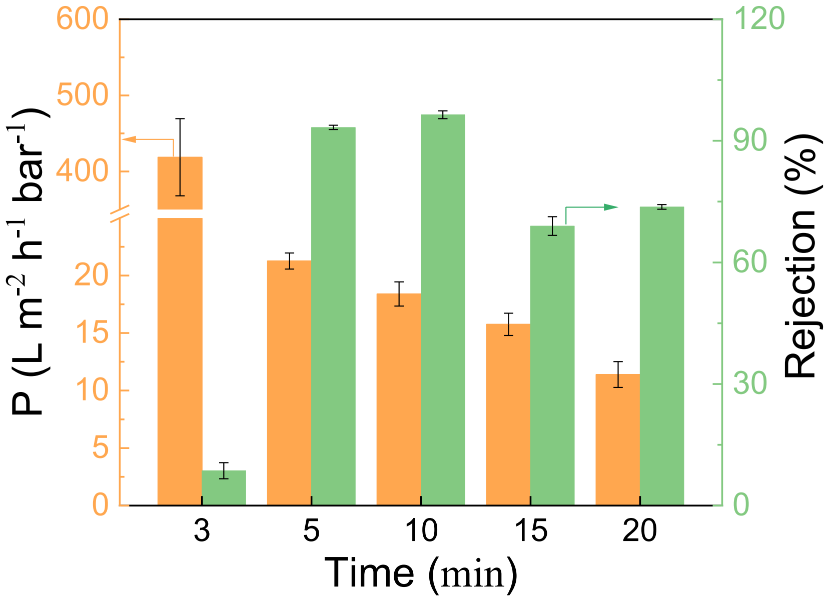


**Supplementary Figure 21.** The effect of volatilization time on NF performance. The error bars represent the standard deviation of data from three replicate measurements.

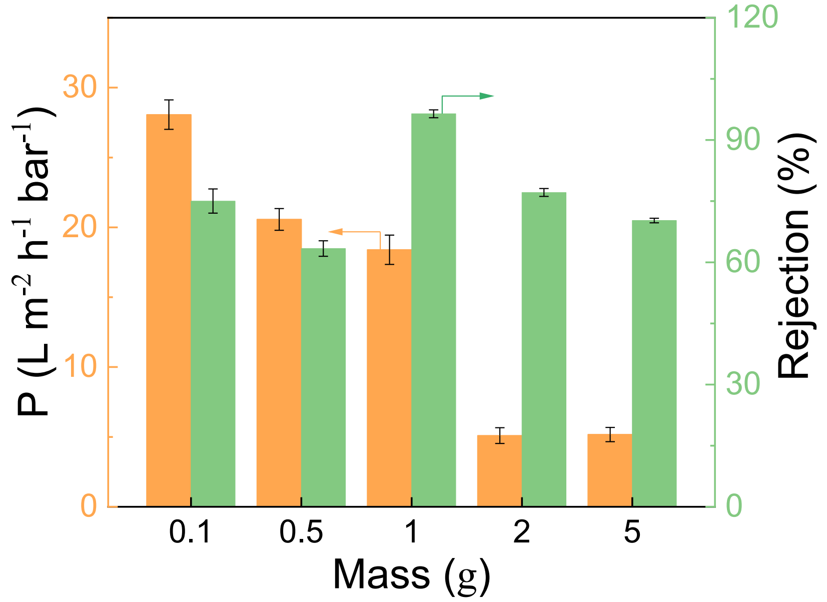


**Supplementary Figure 22.** The effect of volatilization time on NF performance. The error bars represent the standard deviation of data from three replicate measurements.

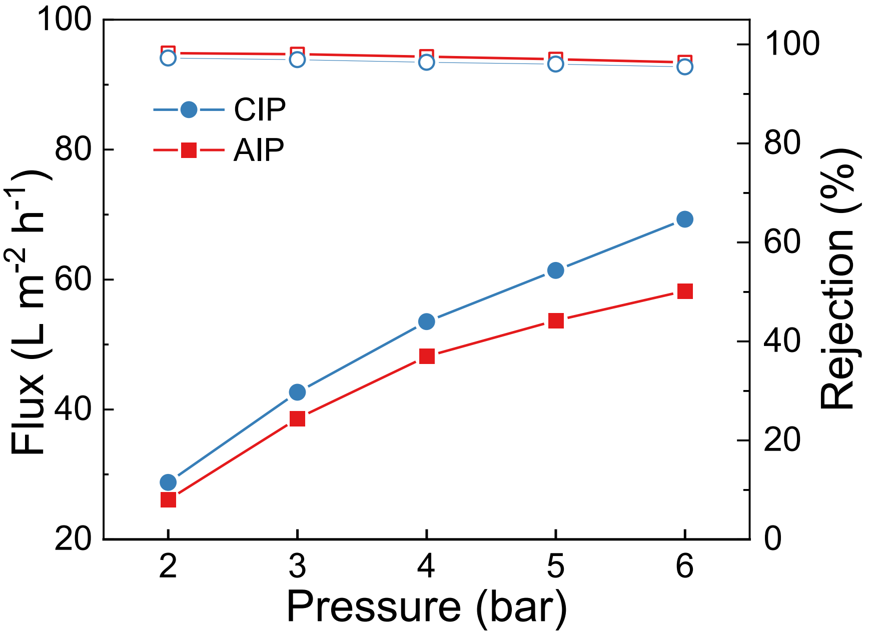


**Supplementary Figure 23.** Flux and rejection of the CIP-PA and AIP-PA NF membranes at different pressure. ($Na_2SO_4$ concentration: 1000 ppm).

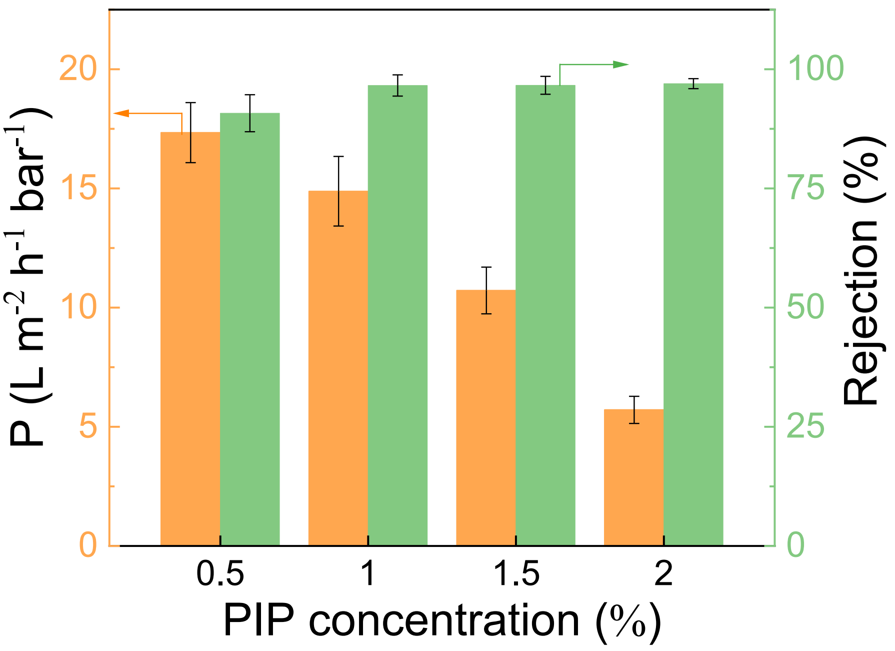


**Supplementary Figure 24.** Effect of PIP concentration on nanofiltration performance of CIP-PA membrane. The error bars represent the standard deviation of data from three replicate measurements.

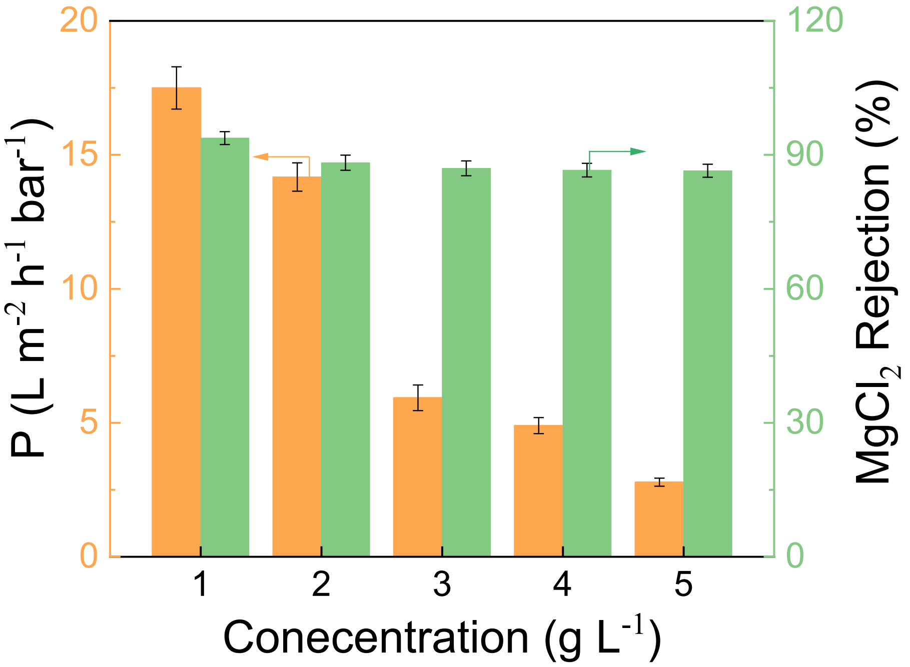


**Supplementary Figure 25.** Effect of $MgCl_2$ concentration on the separation performance of AIP-PA membrane at 4 bar. The error bars represent the standard deviation of data from three replicate measurements.

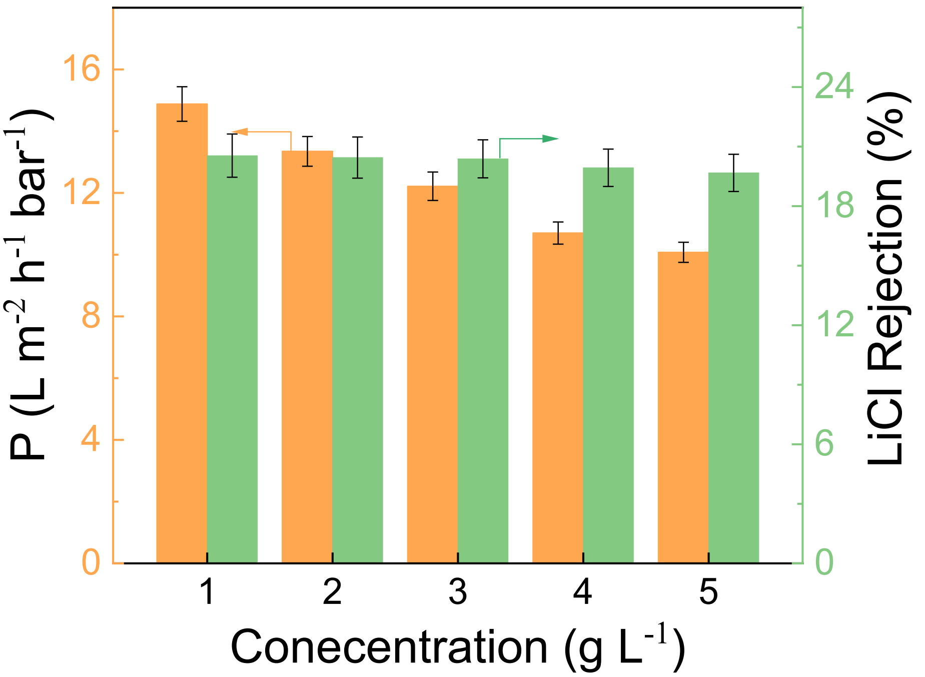


**Supplementary Figure 26.** Effect of LiCl concentration in the separation performance of AIP-PA membrane at 4 bar. The error bars represent the standard deviation of data from three replicate measurements.

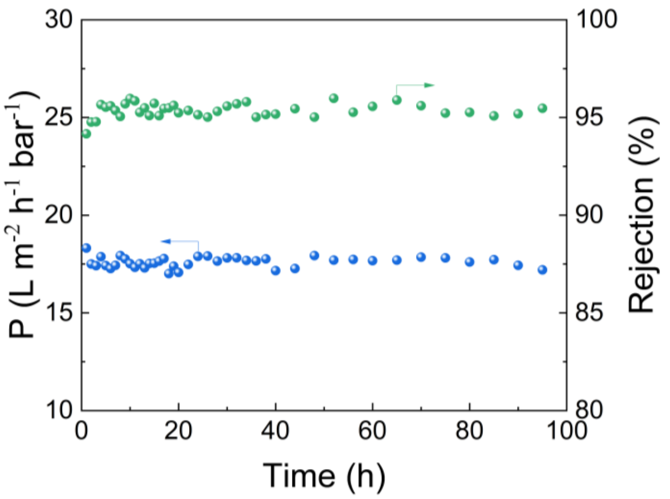


**Supplementary Figure 27.** Permeance and rejection as a function of operation time for AIP-PA membrane tested with a single $MgCl_2$ solution.

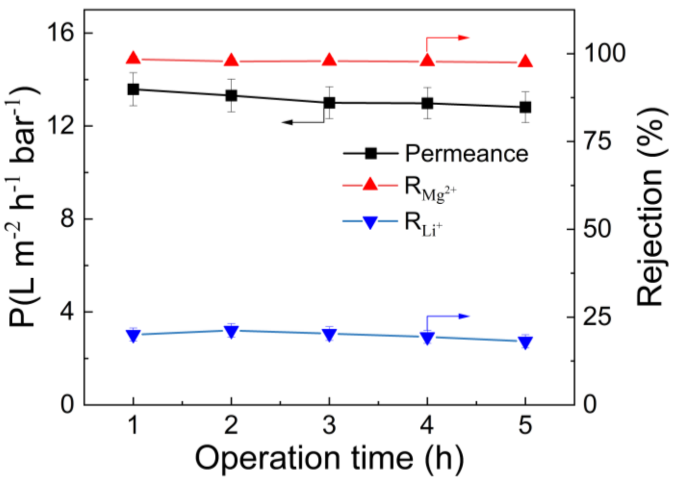


**Supplementary Figure 28.** Permeance and rejection as a function of operation time for AIP-PA membrane tested with a mixture solution (salt concentration: 2000 ppm; $Mg^{2+}/Li^{+}=1$; applied pressure: 4 bar). The error bars represent the standard deviation of data from three replicate measurements.

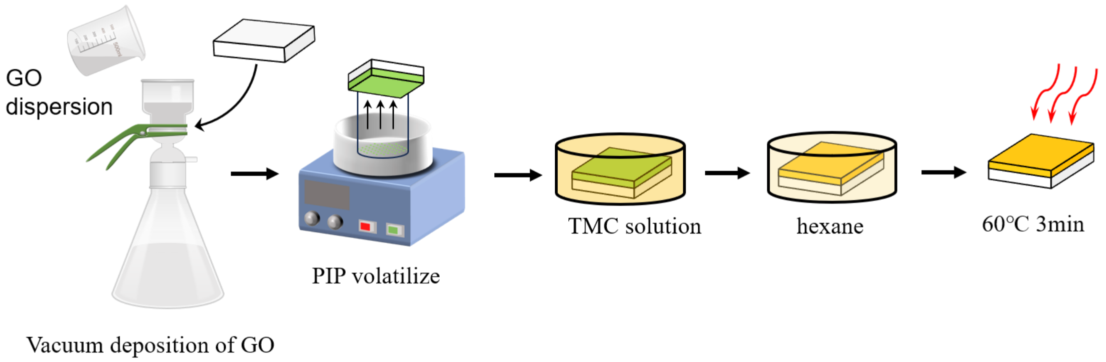


**Supplementary Figure 29.** A schematic illustration showing the preparation process of GL-PA membrane.

**Preparation of GO gutter layer:** Firstly, 1 ml of 2 mg/mL graphene oxide (GO) aqueous solution was accurately prepared, then 1999 mL of deionized water was added. The solution was therefore diluted to be 1 ppm, and then subjected to stirring and sonification for 1 h each. Finally, 20 mL of GO dispersion was taken and filtered onto the surface of PAN substrate by the assistance of vacuum filtration to prepare the GO gutter layer.

**Preparation of GL-PA membrane:** The substrate with GO gutter layer was first dried with filter paper before being placed on a volatilization apparatus. Prior to that, a specific amount of piperazine was added into the volatilization device. Thereafter, the volatilization apparatus was immersed in a temperature-controlled water bath for a period of volatilization. Afterwards, the substrate was taken out and immersed in a 0.2 wv% TMC *n*-hexane solution for 30 s to finish the interfacial polymerization. Finally, the membrane was soaked in an *n*-hexane solution to remove unreacted TMC, and then placed in an oven at 60 °C for 3 minutes before test.

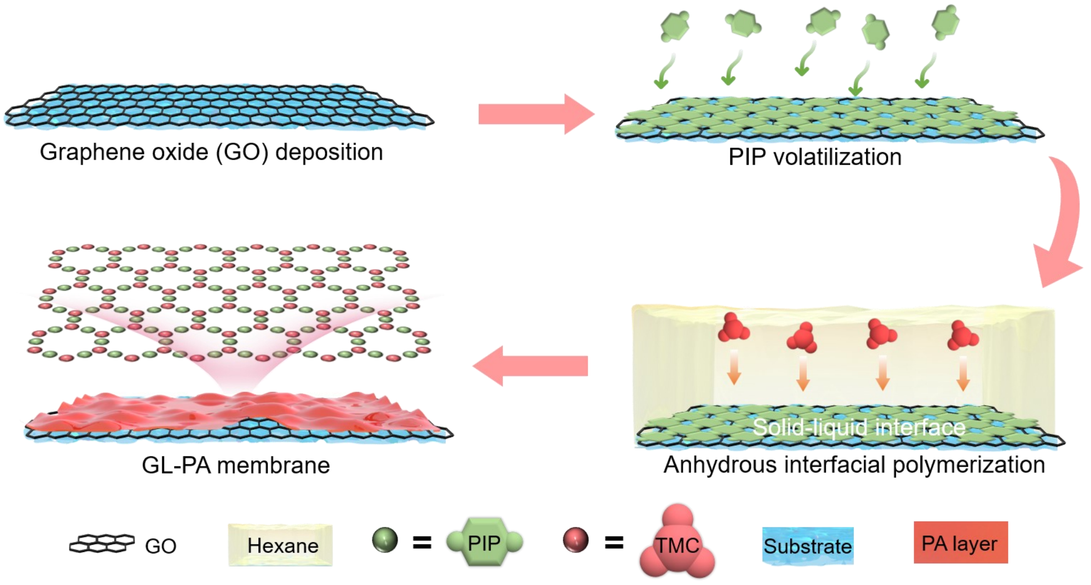


**Supplementary Figure 30.** Scheme depicting the preparation of GL-PA membrane, where PIP, TMC, GL and PA are abbreviations for piperazine, trimesoyl chloride, gutter layer and polyamide, respectively.

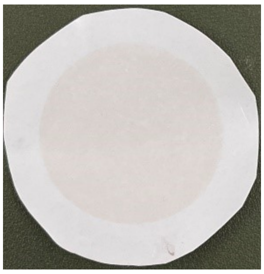

**Supplementary Figure 31.** Photo of GL-PA membrane.

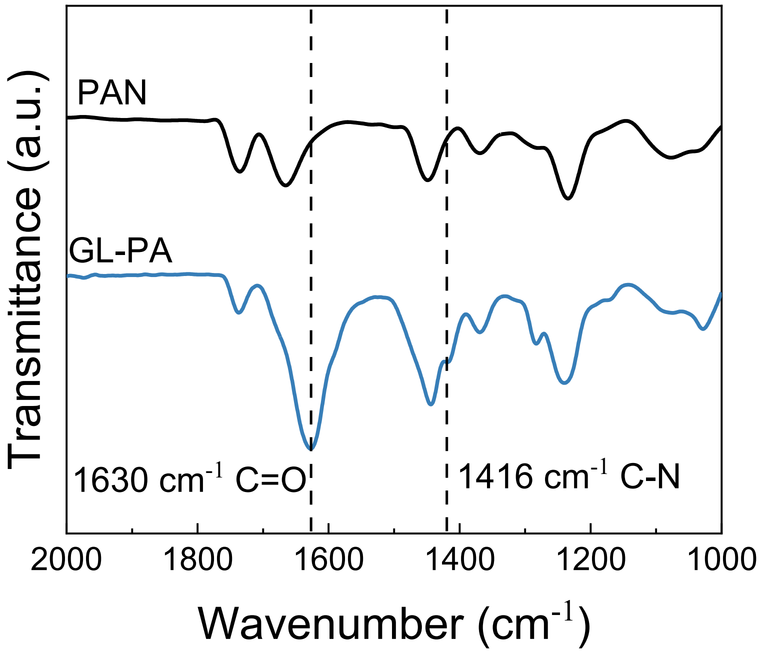


**Supplementary Figure 32.** ATR-FTIR survey of PAN substrate and GL-PA membranes.

As shown in Supplementary Fig. 32, the amide group formed from the reaction between solid PIP and TMC in *n*-hexane is responsible for the new stretching vibration peak of C=O bond at 1630 $cm^{-1}$ and of C-N bond at 1416 $cm^{-1}$, confirming the successful synthesis of the GL-PA layer by AIP.

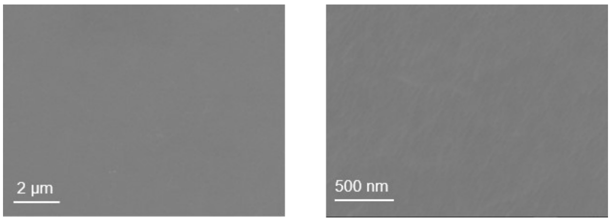


**Supplementary Figure 33.** SEM image of GO deposited on the substrate.

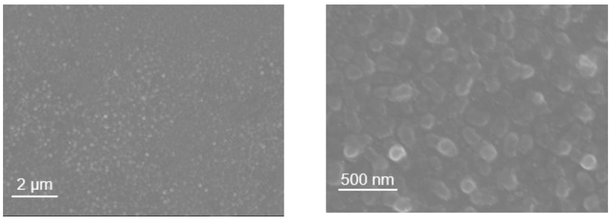


**Supplementary Figure 34.** SEM image of GL-PA membrane.

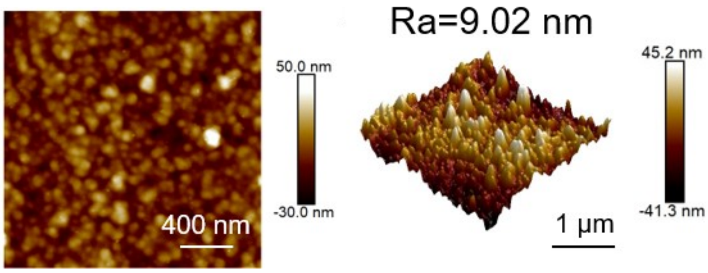


**Supplementary Figure 35.** AFM image of GL-PA membrane.

As shown in Supplementary Fig. 2, compared to the porous PAN substrate (Supplementary Fig.10a), the construction of a GO layer on the PAN substrate resulted in a smoother surface. This gutter layer makes the distribution of volatile PIP molecules more uniform and compact. From Supplementary Fig. 34 and 35, the gutter layer` reduces could avoid the intrusion of PA layer into the pores, and allows PA to grow on the surface of the gutter layer, enhancing the denseness of the polyamide membrane.

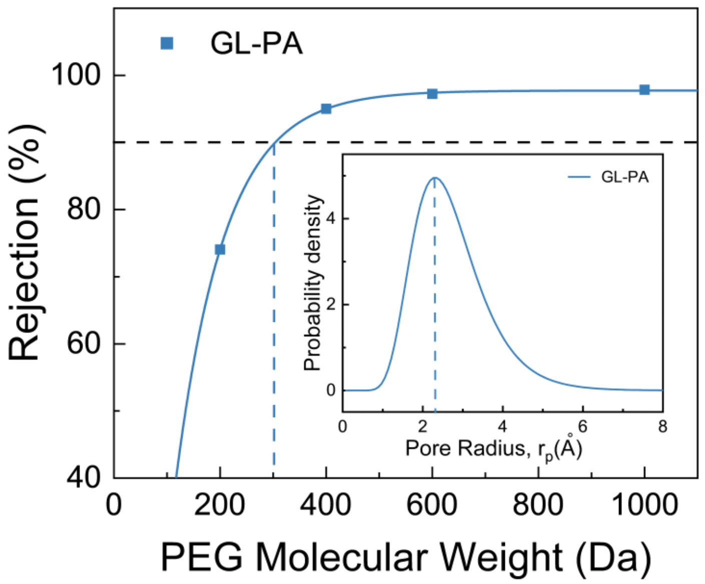


**Supplementary Figure 36.** Rejection of PEG with different molecular weight by GL-PA membrane Inset: pore-size distribution derived from rejection curves of PEG.

The MWCO of the prepared GL-PA membrane is evaluated as 300 Da and the average pore radius is 2.31 Å. Both are smaller than 340 Da and 2.53 Å of AIP-PA membrane. These results prove that the PA membrane prepared with the gutter layer is denser and possibly has a stronger size sieving effect.

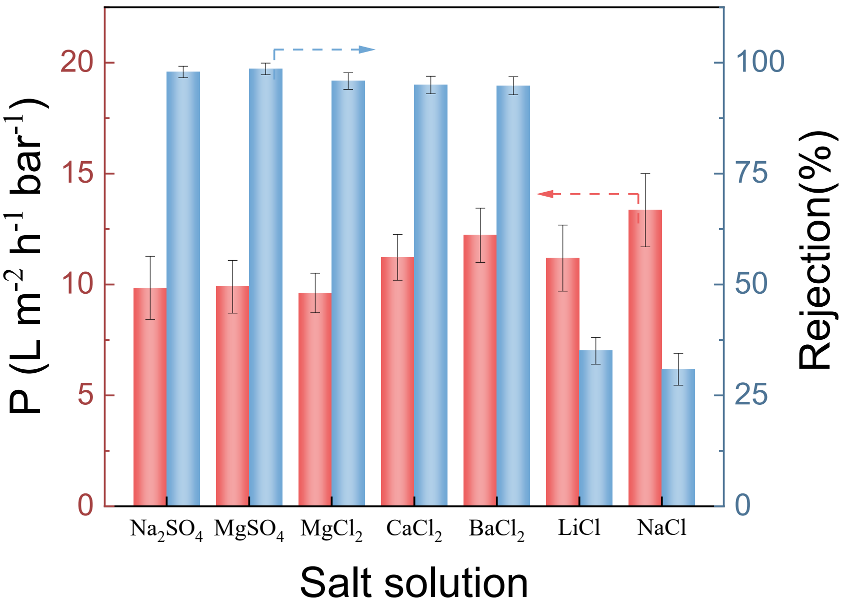


**Supplementary Figure 37.** Salt reject performance of GL-PA membrane. The error bars represent the standard deviation of data from three replicate measurements.

As shown in Supplementary Fig. 37, the rejection of GL-PA membrane for two sulfates were 97.92% ($Na_2SO_4$) and 98.60% ($MgSO_4$), respectively. The retention effect for divalent cationic chloride salts were all above 94%, especially 94.83% for $BaCl_2$, which was slightly higher than 93.59% for the AIP-PA membrane. The rejection of GL-PA membrane for LiCl and NaCl were 35.06% and 30.89%, respectively, which were at least 9% higher than those of AIP-PA membrane. These results imply that the introduction of the interlayer enabled a denser structure with narrower pores inside the resulting PA membrane structure, which was beneficial to the rejection of small molecules.

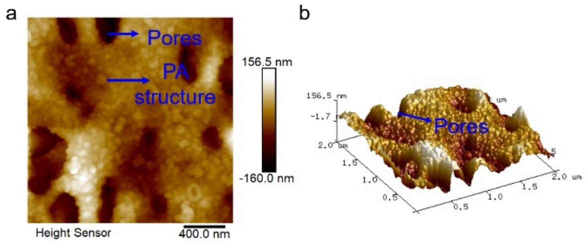


**Supplementary Figure 38.** (a) 2D and (b) 3D AFM images of PA membrane prepared on PES microfiltration substrate. (the pore size of PES substrate: 0.22 μm)

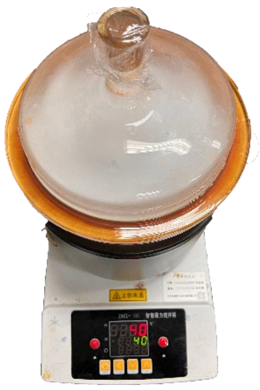

**Supplementary Figure 39.** Large-area AIP-PA membrane preparation device.

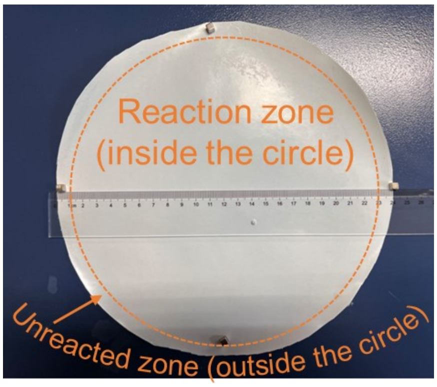


**Supplementary Figure 40.** Photo of large-area AIP-PA membrane.

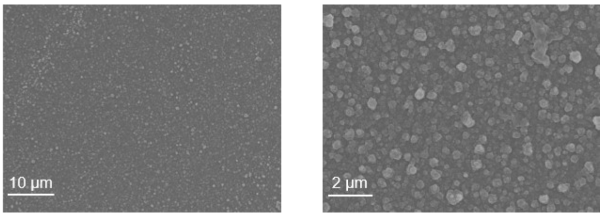


**Supplementary Figure 41.** SEM images of the large-area AIP-PA membrane.

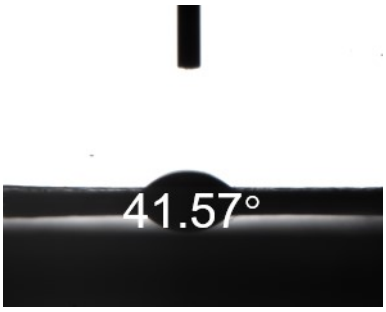


**Supplementary Figure 42.** Water contact angle images of the large-area AIP-PA membrane.

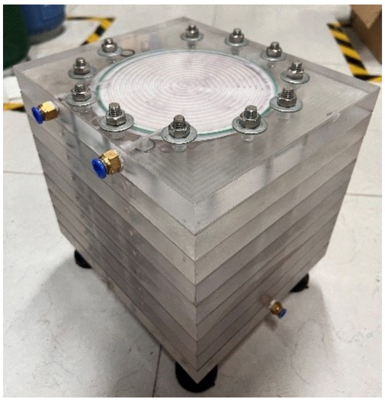

**Supplementary Figure 43.** Photo of large area membrane modules.

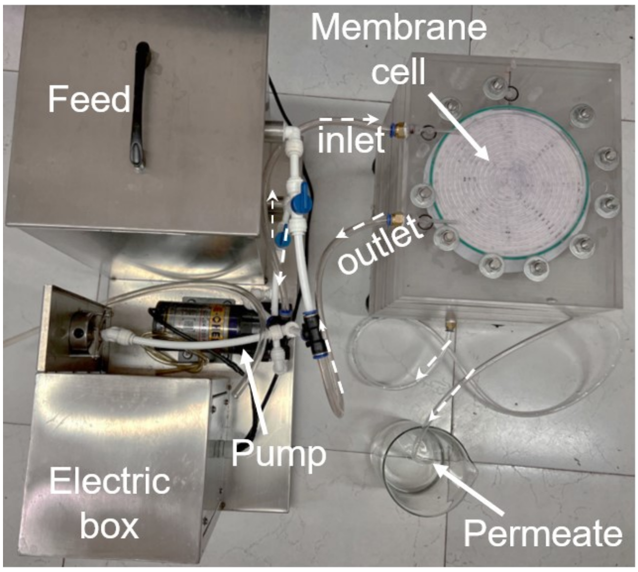


**Supplementary Figure 44.** Photo of the membrane module evaluation system.

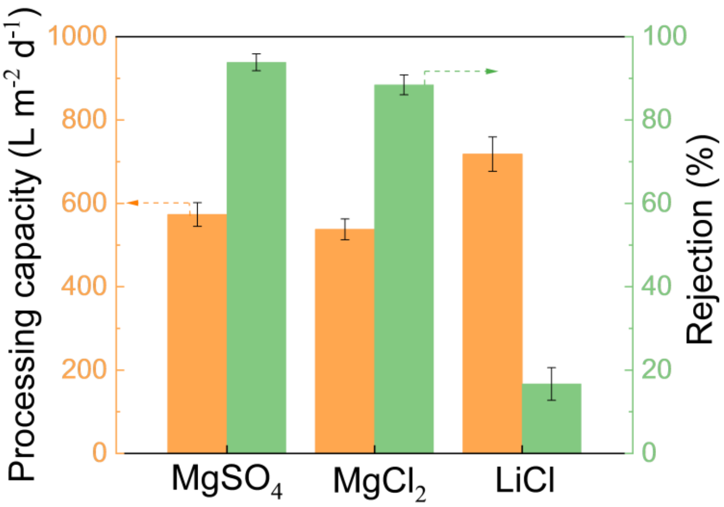


**Supplementary Figure 45.** Salt rejection performance of large-area AIP-PA membrane. The error bars represent the standard deviation of data from three replicate measurements.

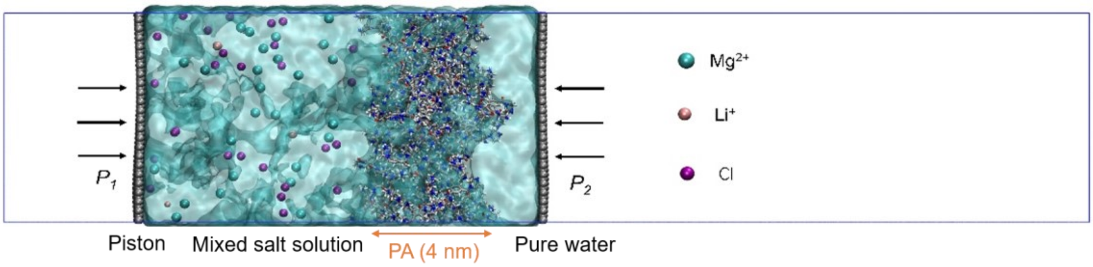


**Supplementary Figure 46.** The MD simulation system with a 4 nm-thick PA membrane.

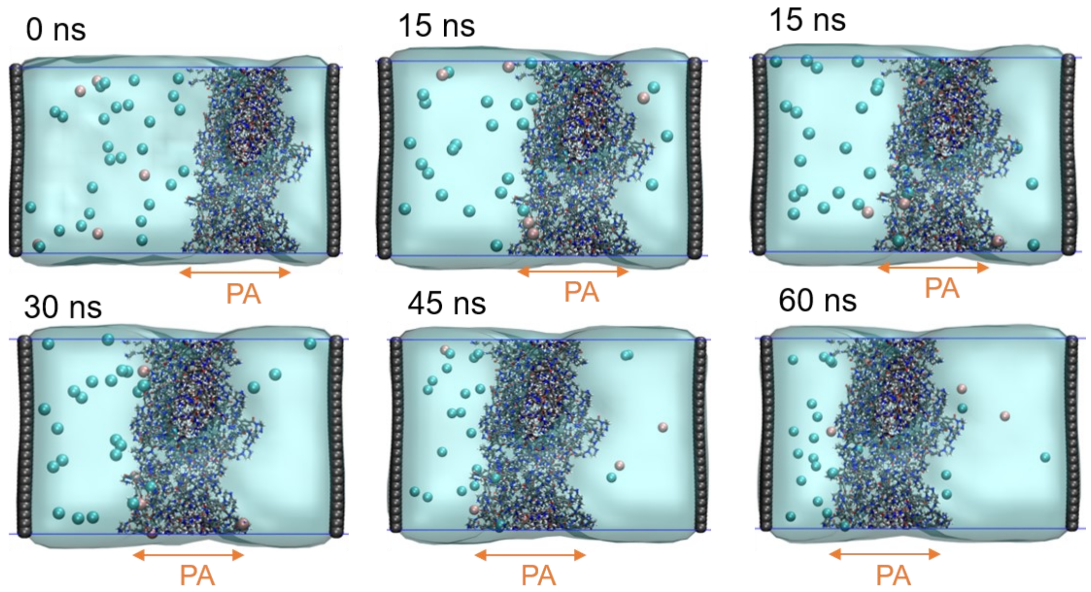


**Supplementary Figure 47.** Simulation system with snapshots at different time for the permeation of Mg and Li ions ($Mg^{2+}/Li^{+}$=30) through the PA membrane, where the blue ball represents $Mg^{2+}$ and the pink ball represents $Li^{+}$.

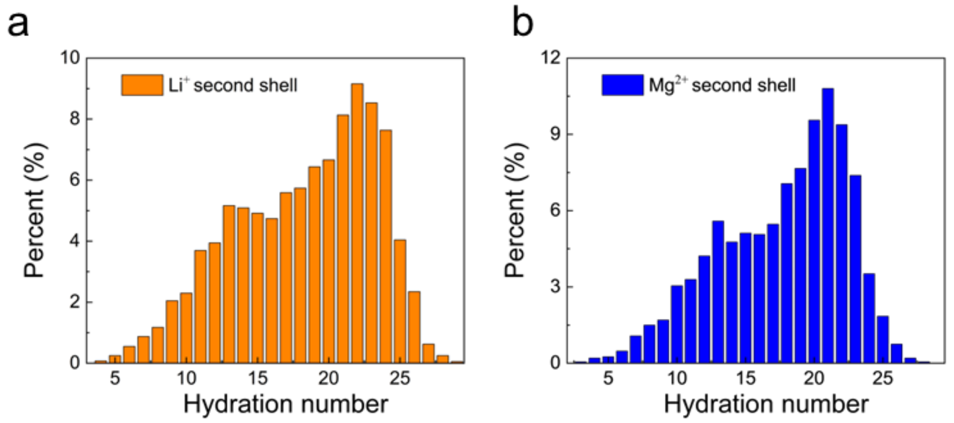


**Supplementary Figure 48.** Hydration number distributions of (a) $Li^{+}$ and (b) $Mg^{2+}$ in the second hydration shell.

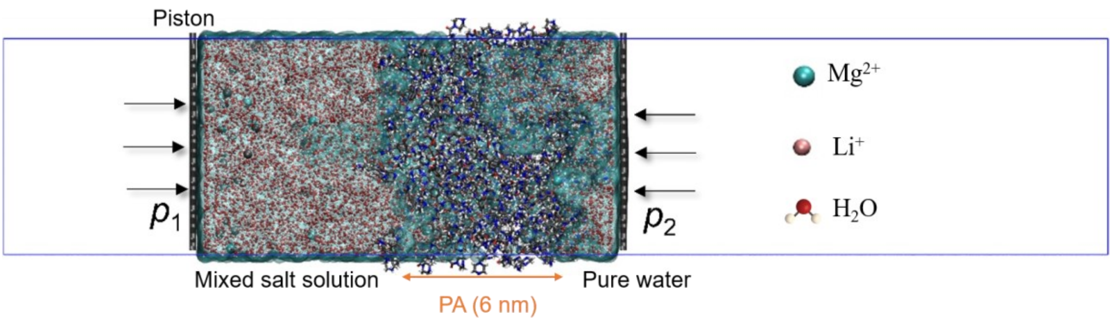


**Supplementary Figure 49.** The calculated simulation system with a 6 nm-thick PA membrane.

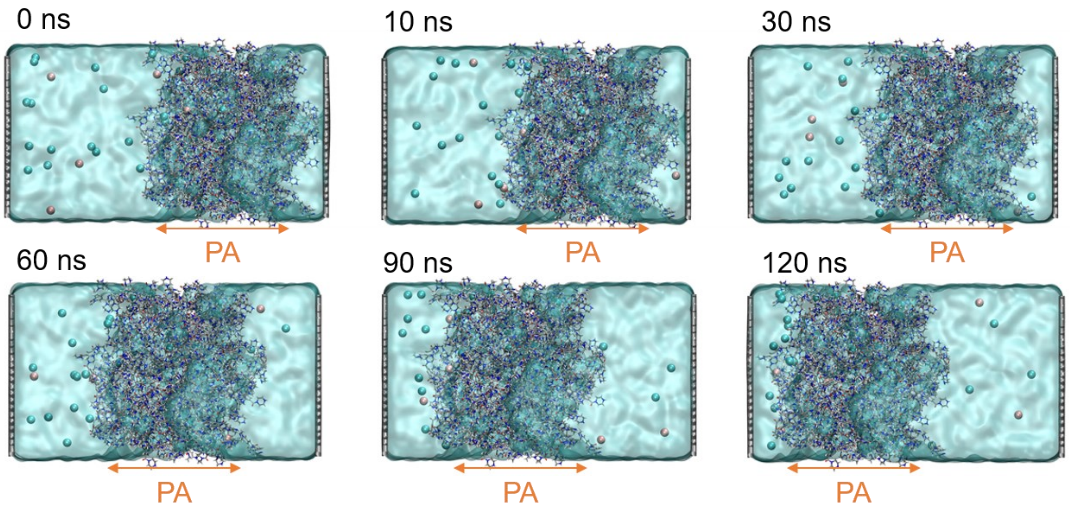


**Supplementary Figure 50.** Image of calculated simulation results with a 6 nm-thick PA membrane, where the blue ball represents $Mg^{2+}$ and the pink ball represents $Li^{+}$.

We also performed one simulation that $Mg^{2+}/Li^{+}$ is about 30 with a membrane thickness of 6 nm (please see Supplementary Figure 49 and 50). The results show that the rejection for $Mg^{+}$ and $Li^{+}$ are about 92% and 33.3%, respectively, which is consistent with our previous simulation and experimental result that the PA membrane shows an excellent Mg/Li selectivity. This result indicates that the size of the membrane used in this work can reflect the real transport process in experiment, to a certain extent.

**Supplementary Table 1.** Elemental compositions and O/N ratio of AIP-PA and CIP-PA Membranes.

| Membrane | C | N | O | O/N |
|---|---|---|---|---|
| PAN | 75.34 | 19.82 | 4.84 | 0.24 |
| CIP-PA | 72.07 | 13.21 | 14.72 | 1.11 |
| AIP-PA@30 | 73.27 | 19.92 | 6.8 | 0.34 |
| AIP-PA@35 | 68.78 | 17.35 | 13.86 | 0.80 |
| AIP-PA@40 | 66.89 | 18.88 | 14.23 | 0.75 |
| AIP-PA@45 | 66.29 | 20.37 | 13.33 | 0.65 |
| AIP-PA@50 | 65.51 | 24.21 | 10.28 | 0.42 |

**Supplementary Table 2.** Results from the XPS high-resolution N (1*s*) spectra of AIP-PA and CIP-PA membranes.

| Membrane | N1*s* | | |
|---|---|---|---|
| | Energy（ev） | Species | (at%) |
| CIP-PA | 399.5 | N-C=O | 97.86% |
| | 401 | C-NH | 2.14% |
| AIP-PA@30 | 399.3 | N-C=O | 2.7% |
| | 398 | CN | 97.3% |
| AIP-PA@35 | 399.3 | N-C=O | 96.63% |
| | 398 | C-NH | 3.37% |
| AIP-PA@40 | 399.32 | N-C=O | 93.50% |
| | 398 | C-NH | 6.50% |
| AIP-PA@45 | 399.34 | N-C=O | 90.41% |
| | 398 | C-NH | 9.59% |
| AIP-PA@50 | 399.3 | N-C=O | 88.18% |
| | 398 | C-NH | 11.82% |

**Supplementary Table 3.** Results from the XPS high-resolution C (1*s*) spectra of AIP-PA and CIP-PA membranes.

| Membrane | C1*s* | | |
|---|---|---|---|
| | Energy（ev） | Species | (at%) |
| CIP-PA | 284.6 | C=C, C-C, C-H | 64.87% |
| | 285.5 | β-shift | 9.81% |
| | 286.1 | C-N(C-N-C=O,C-NH,C-$NH_2^+$) | 11.12% |
| | 287.4 | N-C=O/O-C=O | 14.20% |
| AIP-PA@30 | 283.6 | C-H | 41.76% |
| | 284.6 | C=C, C-C, C-H | 26.40% |
| | 285.2 | C-N | 26.49% |
| | 286.5 | CN | 5.35% |
| AIP-PA@35 | 284.5 | C=C, C-C, C-H | 65.33% |
| | 285.5 | β-shift | 10.91% |
| | 286.1 | C-N(C-N-C=O,C-NH,C-$NH_2^+$) | 7.36% |
| | 287.5 | N-C=O/O-C=O | 16.41% |
| AIP-PA@40 | 284.5 | C=C, C-C, C-H | 62.89% |
| | 285.5 | β-shift | 11.78% |
| | 286.1 | C-N(C-N-C=O,C-NH,C-$NH_2^+$) | 7.99% |
| | 287.5 | N-C=O/O-C=O | 17.33% |
| AIP-PA@45 | 284.5 | C=C, C-C, C-H | 63.31% |
| | 285.5 | β-shift | 10.97% |
| | 286.1 | C-N(C-N-C=O,C-NH,C-$NH_2^+$) | 6.39% |
| | 287.5 | N-C=O/O-C=O | 19.33% |
| AIP-PA@50 | 284.5 | C=C, C-C, C-H | 62.25% |
| | 285.5 | β-shift | 13.23% |
| | 286.1 | C-N(C-N-C=O,C-NH,C-$NH_2^+$) | 5.34% |
| | 287.5 | N-C=O/O-C=O | 19.18% |

**Supplementary Table 4.** Results from the XPS high-resolution O (1*s*) spectra of AIP-PA and CIP-PA membranes.

| Membrane | O1*s* | | |
|---|---|---|---|
| | Energy（ev） | Species | (at%) |
| CIP-PA | 530.65 | N-C=O/O-C=O | 58.13% |
| | 531.5 | N-C=O…H/O-C=O…H | 33.21% |
| | 533 | O-C=O | 4.66% |
| | 537.3 | Absorbed water | 3.99% |
| AIP-PA@30 | 529.75 | N-C=O/O-C=O | 61.73% |
| | 531 | N-C=O…H/O-C=O…H | 24.34% |
| | 532.1 | O-C=O | 13.93% |
| | 537 | Absorbed water | 0.00% |
| AIP-PA@35 | 530.65 | N-C=O/O-C=O | 65.64% |
| | 531.5 | N-C=O…H/O-C=O…H | 27.54% |
| | 533 | O-C=O | 2.16% |
| | 537.2 | Absorbed water | 4.66% |
| AIP-PA@40 | 530.56 | N-C=O/O-C=O | 54.23% |
| | 531.5 | N-C=O…H/O-C=O…H | 41.39% |
| | 533 | O-C=O | 1.41% |
| | 537.2 | Absorbed water | 2.97% |
| AIP-PA@45 | 530.65 | N-C=O/O-C=O | 52.88% |
| | 531.5 | N-C=O…H/O-C=O…H | 40.93% |
| | 533 | O-C=O | 3.00% |
| | 537.2 | Absorbed water | 3.19% |
| AIP-PA@50 | 530.65 | N-C=O/O-C=O | 57.70% |
| | 531.5 | N-C=O…H/O-C=O…H | 35.86% |
| | 533 | O-C=O | 3.12% |
| | 537.2 | Absorbed water | 3.33% |

**Supplementary Table 5.** Ionic radius, hydrated radius, hydration energy, and separation performance of ions investigated in this study.[1–3]

| | Ionic Radius (Å) | Stokes Radius (Å) | Hydrated Radius (Å) | Hydration energy (kcal mol$^{-1}$) | Salt used in the test | Rejection (%) | |
|---|---|---|---|---|---|---|---|
| Cations | | | | | | CIP | AIP |
| $Gd^{3+}$ | 0.94 | 4.08 | 4.59 | 807.4 | $GdCl_3$ | 94.76% | 98.38% |
| $Sm^{3+}$ | 0.96 | 3.98 | 4.54 | 794.3 | $SmCl_3$ | 93.32% | 98.05% |
| $La^{3+}$ | 1.05 | 3.96 | 4.53 | 751.3 | $LaCl_3$ | 93.14% | 97.41% |
| $Co^{2+}$ | 0.72 | 3.35 | 4.23 | 457.7 | $CoCl_2$ | 69.03% | 93.63% |
| $Mg^{2+}$ | 0.65 | 3.47 | 4.23 | 437.4 | $MgCl_2$ | 77.50% | 93.90% |
| $Ca^{2+}$ | 0.99 | 3.1 | 4.12 | 359.7 | $CaCl_2$ | 60.08% | 92.02% |
| $Ni^{2+}$ | 0.7 | 2.92 | 4.04 | 473.2 | $NiCl_2$ | 52.60% | 95.12% |
| $Ba^{2+}$ | 1.35 | 2.92 | 4.04 | 298.8 | $BaCl_2$ | 35.94% | 93.59% |
| $Li^{+}$ | 0.6 | 2.38 | 3.82 | 113.5 | LiCl | 25.38% | 23.53% |
| $Na^{+}$ | 0.95 | 1.84 | 3.58 | 87.2 | NaCl | 21.00% | 21.55% |
| $K^{+}$ | 1.33 | 1.25 | 3.31 | 70.5 | KCl | 20.32% | 20.12% |
| $Rb^{+}$ | 1.48 | 1.18 | 3.29 | 65.7 | RbCl | 15.36% | 15.38% |
| $Cs^{+}$ | 1.69 | 1.19 | 3.29 | 59.8 | CsCl | 13.29% | 13.49% |
| Anions | | | | | | | |
| $SO_4^{2-}$ | 2.9 | 2.3 | 3.82 | 258.1 | $Na_2SO_4$ | 96.59% | 97.32% |
| $Cl^{-}$ | 1.8 | 1.2 | 3.32 | 81.2 | NaCl | 21.00% | 21.55% |

**Supplementary Table 6.** Performance comparison of AIP-PA membrane with reported nanofiltration membranes for $Mg^{2+}/Cl^-$ separation.

| No | Membrane | $C_{total}$ (g/L) | Mg/Li mass ratio | P ($L\ m^{-2}\ h^{-1}\ bar^{-1}$) | SF | Ref |
|---|---|---|---|---|---|---|
| 1 | DAIB | 5.5 | 15.3 | 15.5 | 16.6 | [4] |
| | | 10.5 | 30.6 | 10.8 | 11.1 | |
| | | 25.5 | 76.5 | 3.9 | 5.71 | |
| 2 | PIP-MWCNTS | 2 | 21.4 | 8.5 | 16.4 | [5] |
| 3 | QBPD | 2 | 50 | 16.1 | 5.2 | [6] |
| 4 | LBL coating | 2 | 20 | 10.9 | 81.1 | [7] |
| | | | 40 | 10.6 | 78.5 | |
| | | | 60 | 10 | 87.2 | |
| 5 | PHF-doped | 2 | 21.4 | 6.3 | 13.1 | [8] |
| 6 | MWCNTS-COOK | 2 | 20 | 11.46 | 58.66 | [9] |
| 7 | DK | 10 | 24 | 0.6 | 3.2 | [10] |
| 8 | GO-PA | 2 | 20 | 11.15 | 16.27 | [11] |
| 9 | [MimAP][$Tf_2N$]-PA | 1.1 | 15.3 | 5.7 | 9.57 | [12] |
| | | 2.1 | 30.6 | 4.7 | 8.12 | |
| 10 | MBCN-0.02 | 2 | 73 | 5.6 | 23.9 | [13] |
| 11 | PES/PIP/TMC/PEI | 2 | 20 | 12 | 33.4 | [14] |
| | NF90 | | 20 | 13 | 2.1 | |
| 12 | BPEI/TMC/EDTA | 2.5 | 36.8 | 0.6 | 9.2 | [15] |
| 13 | PEI-TMC | 2 | 20 | 5 | 20 | [16] |
| 14 | PEI/TMC/CNC-COOH | 2 | 30 | 4.2 | 12.2 | [17] |
| | | | 60 | 3.4 | 5.84 | |
| 15 | Desal DL-2540 | 2 | 64 | 7 | 3.3 | [18] |
| 16 | AIP-PA | 2 | 1 | 13.58 | 51.23 | This work |
| | | | 2 | 13.22 | 41.38 | |
| | | | 10 | 12.30 | 66.93 | |
| | | | 30 | 12.28 | 78.27 | |
| | | | 100 | 12.20 | 71.74 | |

**Supplementary Table 7.** Performance comparison of AIP membrane with reported nanofiltration membranes for $Cl^-/SO_4^{2-}$ separation.

| NO | Membrane | #PWP | Rejection (%) | | Separation Factor | Ref. |
|---|---|---|---|---|---|---|
| | | | $Na_2SO_4$ | NaCl | | |
| 1 | 2,5-DABSA + PIP | 20.4 | 97.2 | 37.6 | 22.3 | [19] |
| 2 | PIP+BHTTM | 13.2[a] | 99.5 | 30 | 140.0 | [20] |
| 3 | Seriein+PIP | 16.4 | 97.3 | 32 | 25.2 | [21] |
| 4 | NH2-PEG-NH2+PIP | 5.8 | 99.5 | 58.3 | 83.4 | [22] |
| 5 | PIP+PEI-Dex | 8.6[a] | 91 | 36 | 7.1 | [23] |
| 6 | DCA | 8.3[a] | 98.5 | 12.6 | 58.3 | [24] |
| 7 | SDA+PIP | 5.3[a] | 91 | 73 | 3.0 | [25] |
| 8 | TA + TMC | 23.4[a] | 47 | 15 | 1.6 | [26] |
| 9 | DETA+TMC | 4.5[a] | 60 | 45 | 1.4 | [27] |
| | | 4.7[a] | 52 | 32 | 1.4 | |
| 10 | PHGH+TMC | 2.8[a] | 79.1 | 39 | 2.9 | [28] |
| | | 3.4[a] | 37.4 | 43 | 0.9 | |
| 11 | Biomonoers | 8.14[a] | 93 | 29 | 10.1 | [29] |
| 12 | Macro-porous substrates | 17.8 | 79 | 50 | 2.4 | [30] |
| 13 | PDA coating | 11.4 | 72.5 | 18.4 | 3.0 | [31] |
| | | 3.5 | 44 | 64.1 | 0.6 | |
| 14 | Alumina supports | 1.1 | 94.2[b] | 67.1 | 5.7 | [32] |
| 15 | TA coating | 10.8 | 99 | 23 | 77.0 | [33] |
| 16 | Additive SDS | 17.1 | 99.6 | 27 | 182.5 | [1] |
| 17 | Additive OH-β-CD | 9.8 | 97.8 | 21.9 | 35.5 | [34] |
| 18 | Additive PVA | 13.3[a] | 99.1 | 51.2 | 54.2 | [35] |
| 19 | Additive SDS | 7.5 | 92.3 | 40.48 | 21.5 | [36] |
| 20 | Modifying the active layer with TEO | 17 | 98 | 49.7 | 25.2 | [37] |
| 21 | Modifying the active layer with QAEP | 18.5[a] | 97.8 | 16.1 | 38.1 | [38] |
| 22 | DOW FILMTEC™ NF70 | 7.2 | 97b | 70 | 10.0 | [20] |
| | DOW FILMTEC™ NF90 | 6.7 | 98[b] | 90 | 5.0 | |

| | | | | | | |
|---|---|---|---|---|---|---|
| | GE-Osmonics DL | 10 | 96[b] | 40 | 15.0 | |
| | GE-Osmonics HL | 6.9 | 97[b] | 33 | 22.3 | |
| | Synder NFX | 2.4 | 99[b] | 40 | 60.0 | |
| | Synder NFW | 5.4 | 97[b] | 20 | 26.7 | |
| 23 | Electrostatic-modulated | 35 | 98 | 26.8 | 73.2 | 39 |
| | | 27 | 98 | 28.5 | 90.51 | |
| 24 | Additive salts | 15.7 | 97 | 60.3 | 13.23 | 40 |
| | | 12.5 | 97.5 | 66.6 | 13.36 | |
| | | 13.5 | 96.8 | 56.7 | 13.53 | |
| | | 11.6 | 97.2 | 62.8 | 13.29 | |
| 25 | AIP-PA | 20.32[a] | 97.32 | 21.55 | 29.27 | This work |

[#]PWP: Pure water permeance ($Lm^{-2}h^{-1}bar^{-1}$). [a]Water permeance with NaCl feed was considered as pure water permeance. [b]Rejection of $MgSO_4$.

**Supplementary Table 8.** The Mg and Li concentrations used in simulation and rejection of $Mg^{2+}$ and $Li^+$ at different Mg/Li ratios.

| Mg/Li | Mg<br>m mol/L | Li<br>m mol/L | R ($MgCl_2$) | R (LiCl) |
|---|---|---|---|---|
| 0 | 0 | 471.8 | / | 70.10% |
| 1 | 83.4 | 286.3 | 93.93% | 58.60% |
| 30 | 208.7 | 36 | 91.66% | -33.33% |
| ∞ | 210.1 | 0 | 84.84% | / |

## Supplementary References


1. Liang, Y. *et al.* Polyamide nanofiltration membrane with highly uniform sub-nanometre pores for sub-1 Å precision separation. *Nat. Commun.* **11**, 2015 (2020).

2. Marcus, Y. Thermodynamics of solvation of ions. Part 6.—The standard partial molar volumes of aqueous ions at 298.15 K. *J. Chem. Soc. Faraday Trans.* **89**, 713–718 (1993).

3. Jr, E. R. N. Phenomenological Theory of Ion Solvation. Effective Radii of Hydrated Ions. **63**, 7 (1959).

4. Peng, H. & Zhao, Q. A nano-heterogeneous membrane for efficient separation of lithium from high magnesium/lithium ratio brine. *Adv. Funct. Mater.* **31**, 2009430 (2021).

5. Zhang, H.-Z., Xu, Z.-L., Ding, H. & Tang, Y.-J. Positively charged capillary nanofiltration membrane with high rejection for $Mg^{2+}$ and $Ca^{2+}$ and good separation for $Mg^{2+}$ and $Li^{+}$. *Desalination* **420**, 158–166 (2017).

6. Feng, Y., Peng, H. & Zhao, Q. Fabrication of high performance $Mg^{2+}/Li^{+}$ nanofiltration membranes by surface grafting of quaternized bipyridine. *Sep. Purif. Technol.* **280**, 119848 (2022).

7. He, R. *et al.* Unprecedented $Mg^{2+}/Li^{+}$ separation using layer-by-layer based nanofiltration hollow fiber membranes. *Desalination* **525**, 115492 (2022).

8. Shen, Q., Xu, S.-J., Xu, Z.-L., Zhang, H.-Z. & Dong, Z.-Q. Novel thin-film nanocomposite membrane with water-soluble polyhydroxylated fullerene for the separation of $Mg^{2+}/Li^{+}$ aqueous solution. *J. Appl. Polym. Sci.* **136**, 48029 (2019).

9. Xu, P., Hong, J., Xu, Z., Xia, H. & Ni, Q.-Q. Positively charged nanofiltration membrane based on (MWCNTs-COOK)-engineered substrate for fast and efficient lithium extraction. *Sep. Purif. Technol.* **270**, 118796 (2021).

10. Yang, G., Shi, H., Liu, W., Xing, W. & Xu, N. Investigation of $Mg^{2+}/Li^{+}$ Separation by Nanofiltration. *Chin. J. Chem. Eng.* **19**, 586–591 (2011).

11. Xu, P. *et al.* "Bridge" graphene oxide modified positive charged nanofiltration thin membrane with high efficiency for $Mg^{2+}/Li^{+}$ separation. *Desalination* **488**, 114522 (2020).

12. Wu, H. *et al.* A novel nanofiltration membrane with [MimAP][$Tf_2N$] ionic liquid for utilization of lithium from brines with high $Mg^{2+}/Li^{+}$ ratio. *J. Membr. Sci.* **603**, 117997 (2020).

13. Bi, Q., Zhang, C., Liu, J., Liu, X. & Xu, S. Positively charged zwitterion-carbon nitride functionalized nanofiltration membranes with excellent separation performance of $Mg^{2+}/Li^{+}$ and good antifouling properties. *Sep. Purif. Technol.* **257**, 117959 (2021).

14. Yang, Z. *et al.* Dual-skin layer nanofiltration membranes for highly selective $Li^{+}/Mg^{2+}$

separation. *J. Membr. Sci.* **620**, 118862 (2021).

15. Li, W. *et al.* A positively charged composite nanofiltration membrane modified by EDTA for LiCl/$MgCl_2$ separation. *Sep. Purif. Technol.* **186**, 233–242 (2017).

16. Xu, P. *et al.* Positive charged PEI-TMC composite nanofiltration membrane for separation of $Li^+$ and $Mg^{2+}$ from brine with high $Mg^{2+}/Li^+$ ratio. *Desalination* **449**, 57–68 (2019).

17. Guo, C. *et al.* Ultra-thin double Janus nanofiltration membrane for separation of $Li^+$ and $Mg^{2+}$: "Drag" effect from carboxyl-containing negative interlayer. *Sep. Purif. Technol.* **230**, 115567 (2020).

18. Sun, S.-Y., Cai, L.-J., Nie, X.-Y., Song, X. & Yu, J.-G. Separation of magnesium and lithium from brine using a Desal nanofiltration membrane. *J. Water Process Eng.* **7**, 210–217 (2015).

19. Akbari, A., Aliyarizadeh, E., Mojallali Rostami, S. M. & Homayoonfal, M. Novel sulfonated polyamide thin-film composite nanofiltration membranes with improved water flux and anti-fouling properties. *Desalination* **377**, 11–22 (2016).

20. Tang, Y.-J., Xu, Z.-L., Xue, S.-M., Wei, Y.-M. & Yang, H. A chlorine-tolerant nanofiltration membrane prepared by the mixed diamine monomers of PIP and BHTTM. *J. Membr. Sci.* **498**, 374–384 (2016).

21. Pan, Y. *et al.* Enhanced both perm-selectivity and fouling resistance of poly(piperazine-amide) nanofiltration membrane by incorporating sericin as a co-reactant of aqueous phase. *J. Membr. Sci.* **523**, 282–290 (2017).

22. Tang, Y.-J., Xu, Z.-L., Xue, S.-M., Wei, Y.-M. & Yang, H. Improving the chlorine-tolerant ability of polypiperazine-amide nanofiltration membrane by adding $NH_2$-PEG-$NH_2$ in the aqueous phase. *J. Membr. Sci.* **538**, 9–17 (2017).

23. Bera, A. & K. Jewrajka, S. Tailoring polyamide thin film composite nanofiltration membranes by polyethyleneimine and its conjugates for the enhancement of selectivity and antifouling property. *RSC Adv.* **6**, 4521–4530 (2016).

24. Ren, D., Bi, X.-T., Liu, T.-Y. & Wang, X. Oligo-ethylene-glycol based thin-film composite nanofiltration membranes for effective separation of mono-/di-valent anions. *J. Mater. Chem. A* **7**, 1849–1860 (2019).

25. Rezania, H. (Jafar), Vatanpour, V., Shockravi, A. & Ehsani, M. Study of synergetic effect and comparison of novel sulfonated and carboxylated bulky diamine-diol and piperazine in preparation of negative charge NF membrane. *Sep. Purif. Technol.* **222**, 284–296 (2019).

26. Zhang, Y. *et al.* Composite nanofiltration membranes prepared by interfacial polymerization with natural material tannic acid and trimesoyl chloride. *J. Membr. Sci.* **429**, 235–242 (2013).

27. Chiang, Y.-C., Chang, Y., Chuang, C.-J. & Ruaan, R.-C. A facile zwitterionization in the

interfacial modification of low bio-fouling nanofiltration membranes. *J. Membr. Sci.* **389**, 76–82 (2012).

28. Li, X. *et al.* A novel composite nanofiltration membrane prepared with PHGH and TMC by interfacial polymerization. *J. Membr. Sci.* **466**, 82–91 (2014).

29. Shah, A. A. *et al.* Preparation of highly permeable nanofiltration membranes with interfacially polymerized biomonomers. *J. Membr. Sci.* **627**, 119209 (2021).

30. Yang, H.-C., Wu, M.-B., Hou, J., B. Darling, S. & Xu, Z.-K. Nanofilms directly formed on macro-porous substrates for molecular and ionic sieving. *J. Mater. Chem. A* **6**, 2908–2913 (2018).

31. Wang, T., Qiblawey, H., Sivaniah, E. & Mohammadian, A. Novel methodology for facile fabrication of nanofiltration membranes based on nucleophilic nature of polydopamine. *J. Membr. Sci.* **511**, 65–75 (2016).

32. Amelio, A., Sangermano, M., Kasher, R., Bernstein, R. & Tiraferri, A. Fabrication of nanofiltration membranes via stepwise assembly of oligoamide on alumina supports: Effect of number of reaction cycles on membrane properties. *J. Membr. Sci.* **543**, 269–276 (2017).

33. Yang, X. Controllable interfacial polymerization for nanofiltration membrane performance improvement by the polyphenol interlayer. *ACS Omega* **4**, 13824–13833 (2019).

34. Yao, Z., Guo, H., Yang, Z., Qing, W. & Tang, C. Y. Preparation of nanocavity-contained thin film composite nanofiltration membranes with enhanced permeability and divalent to monovalent ion selectivity. *Desalination* **445**, 115–122 (2018).

35. Tan, Z., Chen, S., Peng, X., Zhang, L. & Gao, C. Polyamide membranes with nanoscale Turing structures for water purification. *Science* **360**, 518–521 (2018).

36. Ang, M. B. M. Y. *et al.* Improved performance of thin-film nanofiltration membranes fabricated with the intervention of surfactants having different structures for water treatment. *Desalination* **481**, 114352 (2020).

37. Yan, F. *et al.* Improving the water permeability and antifouling property of thin-film composite polyamide nanofiltration membrane by modifying the active layer with triethanolamine. *J. Membr. Sci.* **513**, 108–116 (2016).

38. Peng, H., Tang, Q., Tang, S., Gong, J. & Zhao, Q. Surface modified polyamide nanofiltration membranes with high permeability and stability. *J. Membr. Sci.* **592**, 117386 (2019).

39. You, X. *et al.* Electrostatic-modulated interfacial polymerization toward ultra-permselective nanofiltration membranes. *iScience* **24**, 102369 (2021).

40. Shen, L. *et al.* Polyamide-based membranes with structural homogeneity for ultrafast molecular sieving. *Nat. Commun.* **13**, 500 (2022).
